
JOURNAL INFORMATION
==============================
NLM Title Abbreviation: ScientificWorldJournal
Journal ID: ScientificWorldJournal
NLM Journal ID: 101131163
Journal ID: TSWJ

The Scientific World Journal

ISSN: 2356-6140
EISSN: 1537-744X
Publisher: Wiley

ARTICLE INFORMATION
==============================
PMCID: PMC6083978
PMID: 30147576
DOI: 10.1100/tsw.2001.198
Article ID: 317392
Article version: 1
Subjects: Short Report

Requirement of Ecm and Insulin For Maintenance of Liver-Specific Functions and Cell Survival in Primary Rat Hepatocytes

Sidhu Jaspreet S.
Liu Fei
Omiecinski Curtis J. *
Dept. of Environmental Health, University of Washington, Box 354695, Seattle, WA 98195, USA
*Curtis J. Omiecinski: cjo@u.washington.edu
Publication date: 2001
Electronic publication date: 2001 May 1
Volume: 1
First page: 98
Last page: 98
Copyright: Copyright © 2001 Jaspreet S. Sidhu et al.
Copyright year: 2001
License: This is an open access article distributed under the Creative Commons Attribution License, which permits unrestricted use, distribution, and reproduction in any medium, provided the original work is properly cited.
License URL: https://creativecommons.org/licenses/by/3.0/

Funding: National Institute of General Medical Sciences GM-32881

==============================
Miami *Nature Biotechnology* Short Reports
*TheScientificWorld* (2001) 1 (S3), 98SR
ISSN 1532-2246; DOI 10.1100/tsw.2001.198

### REQUIREMENT OF ECM AND INSULIN FOR MAINTENANCE OF LIVER-SPECIFIC FUNCTIONS AND CELL SURVIVAL IN PRIMARY RAT HEPATOCYTES

Jaspreet S. Sidhu, Fei Liu, and Curtis J. Omiecinski*

Dept. of Environmental Health, University of Washington, Box 354695, Seattle, WA 98195, USA
* cjo@u.washington.edu

**INTRODUCTION.** Primary rat hepatocytes are a potentially valuable model system for assessing liver-specific responses to pharmaceutical and toxicological agents. However, many culture renditions exist that are of questionable relevance to hepatocyte biology *in vivo*. Maintenance of liver-specific gene expression indices and patterning of appropriate hepatocyte responsiveness to drug or chemical exposure requires a highly defined and phenotypically stable culture system (1,2,3). In our laboratory's ongoing efforts to better define *in vitro* conditions that accurately reproduce *in vivo* patterns of hepatocyte expression, we have investigated the interplay between the extracellular matrix (ECM) and various trophic factors (4,5). In the present study, we assessed the critical interactions of ECM and insulin in primary rat hepatocytes cultures (6) and evaluated their combined cytoprotective effects. We examined a full range of insulin concentrations, representing fasted, fed, and super-physiological levels of the hormone.

**METHOD.** Primary hepatocytes were isolated from adult rat liver and cultured as previously described (3,6). Briefly, cells were plated on tissue culture treated or on collagen 1, 4 or laminin-coated dishes. A range of dilute concentrations (25–250 μg/ml) of ECM was added as an overlay, 4 h after plating. Cells were also cultured in the presence of varying concentrations of insulin (0,0.01,0.1,1,10,1000 nM) for a 96h period. Cells were treated daily with phenobarbital (0.5 mM; PB) for 24h and total RNA isolated and examined by slot-blot hybridization for cytochrome P450 (CYP) and other markers of liver-specific gene expression. In addition, cell lysates were prepared daily and examined for phosphorylation status of PKB and SAPK/JNK. Accompanying nuclear extracts were prepared for each treatment and examined by EMSA for nuclear mobilization of various transcription factors involved in hepatocyte differentiation, survival and stress response.

**RESULTS.** Dilute ECM additions, even 25-75 μg/ml, enabled preservation of normal hepatocyte morphology and markedly facilitated the response profiles of liver specific genes. Cultivation of primary hepatocytes under low concentrations of insulin (<1 nM) resulted in the maintenance of maximal PB-inducible CYP2B1/2 and 3A1 gene expression for 48-72h post-plating; although 10 nM insulin was required to maintained appropriate induction responses at longer culture intervals. Maintenance of cultures in <1 nM insulin for long culture periods also resulted in compromised morphological integrity of the hepatocytes. The 1 nM insulin concentrations were optimally effective in maintaining CYP2E1 expression up to 96h in culture at levels equivalent to that obtained in freshly isolated hepatocytes. In contrast, levels of the liver-specific genes, albumin and transferrin, were significantly reduced under the restricted insulin concentrations. A pronounced elevation in SAPK/JNK phosphorylation status was observed in primary hepatocytes maintained chronically in the absence of insulin. This activity increased substantially in a time-dependent manner in cells cultured in the absence of ECM overlay and was further associated with a pronounced increase in the recruitment of the AP-1 transcriptional complex. Optimal insulin (10 nM) and ECM (25-75 μg/ml) concentrations were required to maintain the nuclear profile of various liver-specific transcription factors essential for cell differentiation and survival.

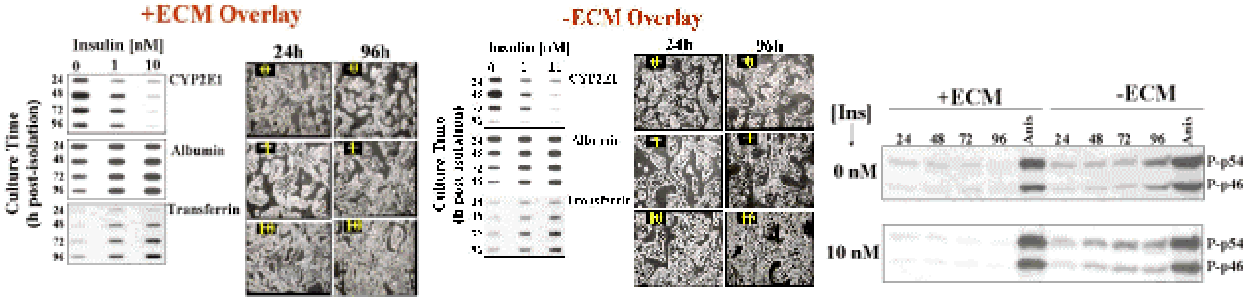

**DISCUSSION.** This study demonstrates the requirement for a highly defined cell culture environment, with defined additions of insulin and ECM, for examining liver-specific responses to xenobiotics, such as PB, as well as for the maintenance of the morphological integrity of hepatocytes. An environment, therefore, favoring both differentiation and survival is proposed. These conditions should better enable accurate prediction of physiologically relevant mechanisms of drug action in culture systems relative to the response *in vivo*.

**ACKNOWLEDGEMENT.** This study was supported by the National Institute of General Medical Sciences (GM-32881) and Burroughs Wellcome Fund

**REFERENCES.**

1. Enat, R., Jefferson, D.M., Ruis-Opazo, N., Gatmaitan, Z., Leinwand, L.A., and Reid, L.M. (1984) Proc. Natl. Acad. Sci. U S A 81, 1411-1415
2. Schuetz, E.G., Li, D., Omiecinski, C.J., Muller-Eberhard, U., Kleinman, H.K., Elswick, B., and Guzelian, P.S. (1988) J. Cell Physiol. 134, 309-323
3. Sidhu, J.S., Farin, F.M., and Omiecinski, C.J. (1993) Arch. Biochem. Biophys. 301, 103-113
4. Zaret, K.S. (1992) Hepatology 15, 1204-1205
5. Rana, B., Mischoulon, D., Xie, Y., Bucher, N.L., and Farmer, S.R. (1994) Mol. Cell. Biol. 14, 5858-5869
6. Sidhu, J.S. and Omiecinski, C.J. (1999) J. Biochem. Mol. Toxicol. 13, 1-9
